# Correlations between the feature of sagittal spinopelvic alignment and facet joint degeneration: a retrospective study

**DOI:** 10.1186/s12891-016-1193-6

**Published:** 2016-08-15

**Authors:** Xin Lv, Yuan Liu, Song Zhou, Qiang Wang, Houyun Gu, Xiaoxing Fu, Yi Ding, Bin Zhang, Min Dai

**Affiliations:** 1 Department of Orthopedics, The First Affiliated Hospital of Nanchang University, No. 17 Yong Wai Zheng Street, Nanchang, Jiangxi 330006 China; 2 Artificial Joints Engineering and Technology Research Center of Jiangxi Province, Nanchang, Jiangxi 330006 China

**Keywords:** Pelvic incidence, Facet joint degeneration, Lumbar lordosis, Sagittal spinopelvic balance

## Abstract

**Background:**

Sagittal spinopelvic alignment changes associated with degenerative facet joint arthritis have been assessed in a few studies. It has been documented that patients with facet joint degeneration have higher pelvic incidence, but the relationship between facet joint degeneration and other sagittal spinopelvic alignment parameters is still disputed. Our purpose was to evaluate the correlation between the features of sagittal spinopelvic alignment and facet joint degeneration.

**Methods:**

Imaging data of 140 individuals were retrospectively analysed. Lumbar lordosis, pelvic tilt (PT), pelvic incidence (PI), sacral slope, and height of the lumbar intervertebral disc were measured on lumbar X-ray plates. Grades of facet joint degeneration were evaluated from the L2 to S1 on CT scans. Spearman’s rank correlation coefficient and Student’s t-test were used for statistical analyses, and a *P*-value <0.05 was considered statistically significant.

**Results:**

PI was positively associated with degeneration of the facet joint at lower lumbar levels (*p* < 0.001 *r* = 0.50 at L5/S1 and *P* = 0.002 *r* = 0.25 at L4/5). A significant increase of PT was found in the severe degeneration group compared with the mild degeneration group: 22.0° vs 15.7°, *P* = 0.034 at L2/3;21.4°vs 15.1°, *P* = 0.006 at L3/4; 21.0° vs 13.5°, *P* = 0.000 at L4/5; 20.8° vs 12.1°, *P* = 0.000 at L5/S1.

**Conclusion:**

Our results indicate that a high PI is a predisposing factor for facet joint degeneration at the lower lumbar spine, and that severe facet joint degeneration may accompany with greater PT at lumbar spine.

**Electronic supplementary material:**

The online version of this article (doi:10.1186/s12891-016-1193-6) contains supplementary material, which is available to authorized users.

## Background

Proper sagittal alignment could enable individuals to maintain a standing posture with a minimum expenditure of energy [1–3]. Loss of normal spinopelvic alignment may accelerate degeneration of motion segments [4]. The motion segment is composed of three articulations between adjacent vertebrae: two facet joints and one disc. A series of studies investigated the correlation of sagittal alignment with lumbar disc degenerative diseases [4–6]. Most researchers have treated the facet joint as a part of the spine, analysing the whole spine by dividing it into conditions with or without degeneration. Relatively few studies have examined the features of spinopelvic alignment in patients with lumbar facet joint degeneration [7, 8].

However, the correlation between sagittal alignment and lumbar facet joint degeneration is still unclear. Therefore, we performed this study to clarify the relationship between the degree of lumbar facet joint degeneration and spinopelvic sagittal parameters.

## Methods

The study was approved by the Ethics Committee of the First Affiliated Hospital of Nanchang University, China. We included and retrospectively analysed lumbar x-ray plates and computed tomography (CT) scans of 140 individuals (with a median age of 34.92, 560 functional units between the L2 and S1) who presented to our hospital between June 2014 and November 2014. The inclusion criteria were (1) age between 30 and 40, (2) no history of spine or pelvic surgery, (3) no spinal deformity, (4) and no spinal fractures, infection, spinal tuberculosis, or other lesions that may have changed the sagittal alignment.

Assessment of face joint degeneration was based on the grading scale described by Pathria. Grade 0 (normal) indicates normal facet joints, whereas grades 1 to 3 show increasing signs of facet joint degeneration with each grade, including signs of the lower one. Grade 1 displays joint space narrowing, grade 2 shows sclerosis, and grade 3 demonstrates osteophytes [9].

**Fig. 1:**
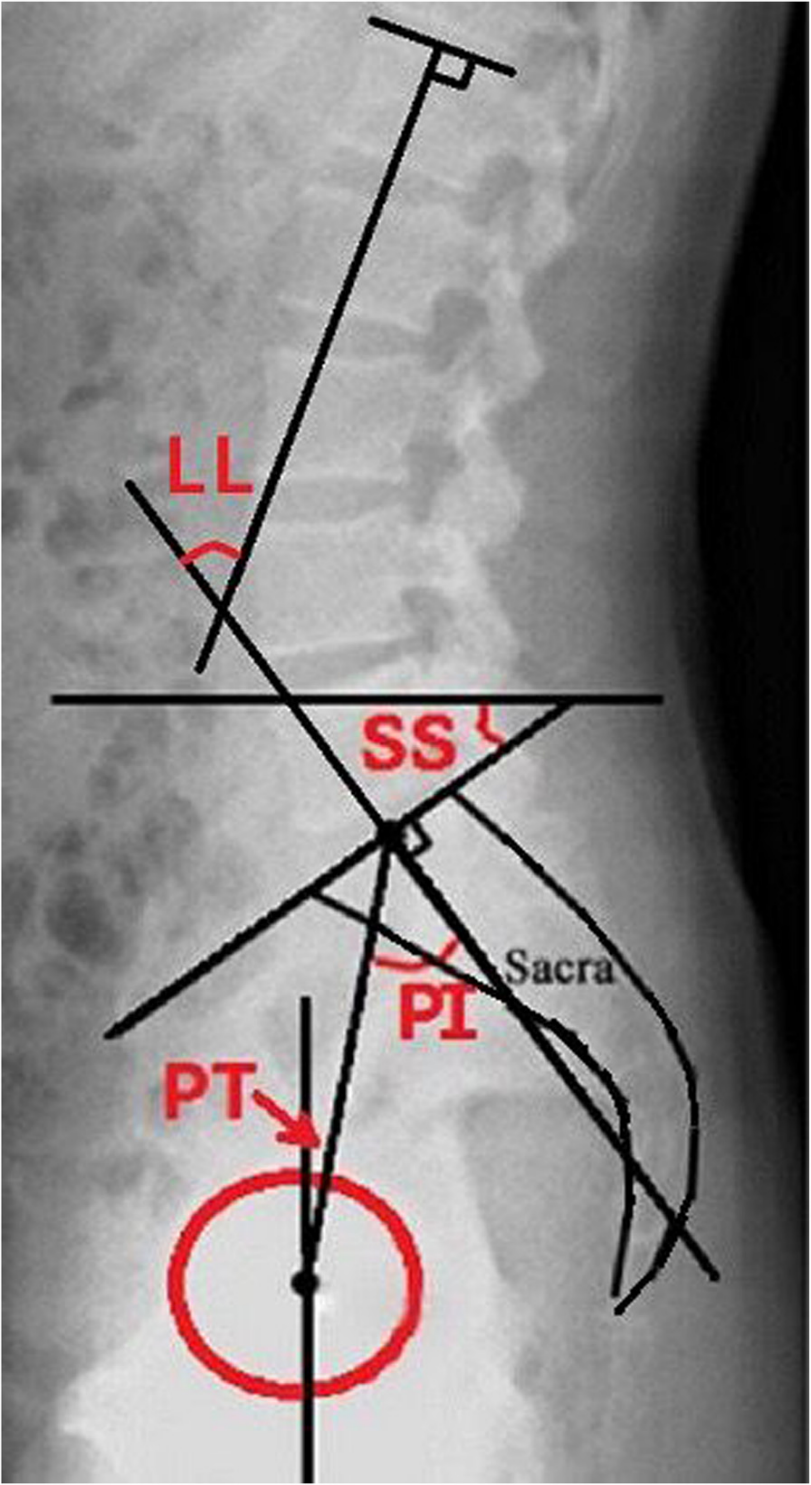
Measurement of Spino-pelvic sagittal parameters. Lumbar lordosis (LL) was measured using the Cobb angle between the superior endplate of the L1 and S1. The pelvic tilt angle (PT) was defined as the angle between a straight line connecting the midpoint of the bilateral femoral head centre to the midpoint of the sacral plate and the plumb line. The pelvic incidence angle (PI) was defined as the angle between the perpendicular line of the sacral plate and the line of the midpoint of the superior endplate of S1 joining with the center of the hip axis. The sacral slope (SS) was defined as the angle formed by the upper endplate of S1 and the horizontal plane

**Fig. 2:**
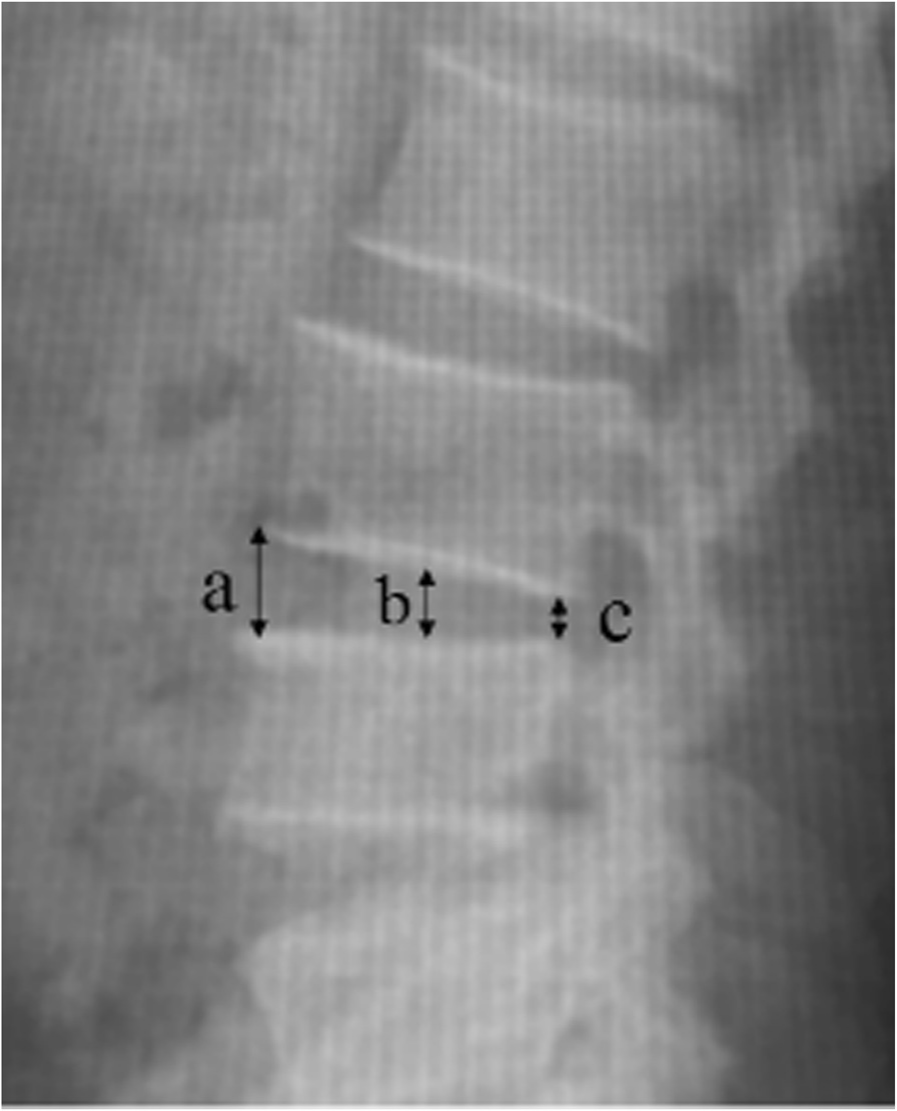
Measurement of height of lumbar intervertebral disc. The height of the lumbar intervertebral disc was calculated by dividing the sum of the heights of the anterior, middle, and posterior intervertebral discs by 3 (a + b + c/3)

Spinopelvic sagittal parameters were measured on a lateral lumbar spine X-ray; lumbar lordosis (LL) was measured using the Cobb angle between the superior endplate of the L1 and S1. The pelvic tilt angle (PT) was defined as the angle between a straight line connecting the midpoint of the bilateral femoral head centre to the midpoint of the sacral plate and the plumb line. The pelvic incidence angle (PI) was defined as the angle between the perpendicular line of the sacral plate and the line of the midpoint of the superior endplate of S1 joining with the center of the hip axis. The sacral slope (SS) was defined as the angle formed by the upper endplate of S1 and the horizontal plane (Fig. 1). The height of the lumbar intervertebral disc was calculated by dividing the sum of the heights of the anterior, middle, and posterior intervertebral discs by 3 (a + b + c/3) (Fig. 2).

All radiographical evaluation was performed by two residents using the PACS system (Picture Archiving and Communication System, and the grade of facet joint degeneration was determined as the side with severe degeneration. Spinopelvic parameters take the average of the two measurements.

Statistical analysis was performed using the SPSS17.0 software. As the PI is constant in adulthood, facet joint degeneration is an ordinal measure. Spearman’s rank correlation coefficient was calculated to investigate the association between increased PI and facet joint degeneration. All individuals were classified into one of two groups according to the facet joint degeneration degree. Group A had mild degeneration (grades 0–1) and Group B had severe degeneration (grades 2–3), Statistical analyses were performed with Student’s t-test to compare the differences of sagittal spinopelvic alignments among groups. A *P*-value <0.05 was deemed significant.

## Results

PI showed a significant association with facet joint degeneration at lower lumbar levels (*p* < 0.001 *r* = 0.50 at L5/S1 and *P* = 0.002 *r* = 0.25 at L4/5). Individuals who had an increased PI were more prone to a degree of higher facet joint degeneration at these levels. However, PI did not correlate with facet joint degeneration at the other lumbar levels (*p* = 0.455 and 0.1 at L2/3 and L3/4).

No significant differences were found between the mild degeneration group and severe degeneration group in terms of age gender, and height of the lumbar intervertebral disc at all levels (Table 1). The radiographic spinopelvic alignment of all segments are detailed in Table 2. The analysis showed a significant increase of PT in the severe degeneration group compared to the mild degeneration group: 22.0° vs 15.7°, *P* = 0.034 at L2/3; 21.4° vs 15.1°, *P* = 0.006 at L3/4; 21.0° vs 13.5°, *P* = 0.000 at L4/5; 20.8° vs 12.1°, *P* = 0.000 at L5/S1. However, the mean SS and LL values for the two groups yielded no significant differences in any levels.

**Table 1:** Demographic comparison of the two groups at baseline

	Group A	Group B	*P*-value
No. of subjects (n)			
L2/3	125	15	-
L3/4	112	28	-
L4/5	86	54	-
L5/S1	71	69	-
Age (years)			
L2/3	34.99 ± 3.01	34.33 ± 3.09	0.427
L3/4	35.08 ± 3.00	34.29 ± 3.06	0.214
L4/5	34.60 ± 3.00	35.43 ± 3.00	0.118
L5/S1	34.55 ± 2.95	35.30 ± 3.06	0.140
Gender (Female:Male)			
L2/3	68:57	7:8	0.570
L3/4	60:52	15:13	1.000
L4/5	46:40	29:25	0.980
L5/S1	39:32	36:33	0.743
Height of lumbar intervertebral disc (mm)			
L2/3	9.70 ± 0.50	9.54 ± 0.41	0.252
L3/4	9.69 ± 0.44	9.65 ± 0.67	0.687
L4/5	9.91 ± 0.46	9.89 ± 0.61	0.843
L5/S1	10.06 ± 0.480	9.96 ± 0.46	0.215

**Table 2:** Comparison of the two groups based on LL, PT, and SS

	Group A	Group B	*P*-value
LL (°)			
L2/3	43.4 ± 14.6	42.1 ± 16.5	0.734
L3/4	43.0 ± 15.5	43.7 ± 13.8	0.923
L4/5	43.2 ± 13.9	43.4 ± 16.0	0.931
L5/S1	41.6 ± 14.0	45.0 ± 15.4	0.171
PI (°)			
L2/3	48.1 ± 11.2	52.7 ± 14.4	0.155
L3/4	47.6 ± 11.5	52.6 ± 11.7	0.042^a^
L4/5	45.9 ± 9.9	53.0 ± 12.8	0.000^a^
L5/S1	43.5 ± 10.4	53.9 ± 10.5	0.000^a^
PT (°)			
L2/3	15.7 ± 10.6	22.0 ± 12.1	0.034^a^
L3/4	15.1 ± 10.6	21.4 ± 11.2	0.006^a^
L4/5	13.5 ± 9.3	21.0 ± 11.8	0.000^a^
L5/S1	12.1 ± 9.4	20.8 ± 10.7	0.000^a^
SS (°)			
L2/3	32.4 ± 10.4	30.7 ± 12.9	0.538
L3/4	32.5 ± 11.0	31.2 ± 9.1	0.574
L4/5	32.4 ± 10.4	32.0 ± 10.1	0.862
L5/S1	31.4 ± 10.7	33.1 ± 10.6	0.346

*Group A* mild degeneration group, grade 0–1, *Group B* severe degeneration group, grade 2–3, *LL* lumbar lordosis, *PT* pelvic tilt, *SS* sacral slope

^a^Means significant difference

## Discussion

In contrast to numerous discussions about the relationship between spinopelvic parameters and degenerative disc disease, only a few studies have investigated the correlation between sagittal alignment and facet joint degeneration. Considering that facetogenic pain is a non-negligible cause of low back pain [10–12], assessing the factors associated with it, such as specific alterations in spinal and sacral parameters, might prove invaluable.

### PI; a potential cause of degenerative processes

PI was first described by During in 1985 [13]. It maintains a constant value in adulthood, is unaffected by posture or position [1, 14], and determines the pelvic orientation, which is represented by the SS and PT, as well as LL. Therefore, the PI plays a basic role in sagittal spinopelvic alignment [1, 14, 15].

In the present study, individuals with severe facet joint degeneration were found to have a higher PI value. However, comparison of PI with facet joint degeneration in the upper lumbar spine did not show any significant differences. This is similar to the findings of a recent study by Jentzsch et al. [7], who found that increased PI may lead to arthritis of the facet joints at lower lumbar spine.

Degenerative spondylolisthesis is characterized by the slipping of one vertebra over another with an intact neutral arch, and is presumed to result from the failure of a locking mechanism caused by facet joint degeneration [16]. PI was found to be significantly greater in patients with spondylolisthesis compared to reference groups [17, 18]. A linear association between PI and spondylolisthesis was found by Labelle et al. [19]. Patients with increased PI are more likely to have a higher risk of degenerative spondylolisthesis. Increased PI leads to higher mechanical stress on the lumbar facet joints [19]. Meanwhile, the lower lumbar facet joints carry the highest loads [20]. High stress on the facet joint can induce lumbar facet joint degeneration [21]. Therefore, greater PI may result in degeneration of the lower facet joints.

Therefore, patients with increased PI are more likely to present with facet joint arthritis and possibly associated back pain. Once these patients with increased PI become symptomatic, orthopaedic surgeons may consider facet joint infiltration and establishing less lordosis with percutaneous instrumentation, where available, in order to restore the spinopelvic balance and prevent facet joint arthritis if they feel that this may cause problems for the patient. If a lumbar spine fracture patient with a large PI needs spinal surgery, spondylodesis may be preferred over percutaneous instrumentation because these patients are more likely to suffer from facet joint arthritis and its related pain.

### PT and LL

A posterior tilt of the pelvis is usually compensation for the anterior displacement of one’s center of gravity. Zhu et al. [22] reported an average of 11.2° of PT in asymptomatic individuals. Compared to this value, in our severe facet joint degeneration group, the average PT was relatively high. Barrey et al. [5] compared the spinopelvic alignment of 32 patients with degenerative disc disease with that of 154 asymptomatic adults and found increased PT in patients with degenerative disc disease. It seems contradictory to our result that patients with severe facet joint degeneration showed larger PT. However, considering that retroversion is the only compensatory mechanism in the pelvis area, the global capacity of pelvis retroversion is determined by PI, and it can be easily achieved for individuals with a high PI. Thus, we speculated that increased PT in the severe degeneration group might be due to compensation for severe degeneration of the facet joint. The backward tilt of the pelvis relieves the contact force exerted on the facet joint.

Nearly 75 % of LL is contributed by levels L4-S1, while the lowest three facet joints were shown to carry the highest loads. Therefore, hyperlordosis is very likely to lead to severe pathology of the lower lumbar facet joints. However, our result did not reveal any relationship between LL and facet joint degeneration. Similarly, Papadakis et al. [23] found no association between LL and facet joint arthritis in 112 females. In addition, Lin et al. [24] reported no significant differences in LL between groups with and without spinal degenerative changes. Conversely, Jentzsch et al. [8] suggested that hyperlordosis is associated with facet joint degeneration at the lower lumbar spine. When we attempted to compare our results with those of previous studies, we found that even when Cobb’s method was used, different authors choose different start and endplate in measurements. Another problem is that the method of grouping varied. This lack of standardization between studies causes difficulty in making exact comparisons. Due to the retrospective nature of our study, we were not able to confirm the role LL plays in facet joint degeneration.

### Limitations

However, there are some limitations in this retrospective study. We did not include whole-spine measurement using cervical and thoracic images, which is equally important for spinopelvic balance. Another defect of retrospective study was that it was difficult to exclude the influence of muscle condition. What is more, we used the purely radiographic assessment of facet joint degeneration using the Pathria grading scale, which may overlook the degeneration of articular cartilage [25], instead of also using clinical parameters. Therefore, further detailed and longitudinal prospective analysis is required.

## Conclusion

The anatomic orientation of the pelvis with a high incidence seems to represent a predisposing factor for facet joint degeneration at lower lumbar spine. Severe facet joint degeneration may be accompanied by greater PT at lumbar spine.

## Abbreviations

LL, Lumbar lordosis; PACS system, picture archiving and communication systems; PI, pelvic incidence; PT, pelvic tilt; SS, sacral slope

## Additional file

Additional file 1: The dataset supporting the conclusions of this article. (XLSX 22 kb)
